# Structural Entropy, Modal Diversity Entropy, and Accessibility Differentiation: A Study of the Western China–Central Asia Cross-Border Multimodal Transportation Network

**DOI:** 10.3390/e28070823

**Published:** 2026-07-20

**Authors:** Ruifen Sun, Ying Xin, Yang Shao, Peilun Ju

**Affiliations:** 1 School of Modern Posts (Logistics), Xi’an University of Posts and Telecommunications, Xi’an 710061, China; sunruifen@xupt.edu.cn (R.S.); xinying@stu.xupt.edu.cn (Y.X.); 2 School of Economics and Management, Chang’an University, Xi’an 710064, China; jupeilun@chd.edu.cn

**Keywords:** Western China–Central Asia, cross-border integrated transportation network, structural entropy, modal diversity entropy, complex network, community structure, accessibility

## Abstract

Cross-border multimodal transportation systems are essential for regional connectivity, yet their structural concentration, modal imbalance, community organization, and accessibility differentiation remain insufficiently understood from an entropy perspective. Based on 2024 data, this study constructs railway, highway, aviation, and integrated transportation networks between Western China and the five Central Asian countries. It integrates complex network analysis, structural entropy, modal diversity entropy, Louvain community detection, and accessibility assessment within a structure–organization–function framework. The results reveal a core–periphery pattern, with Xi’an, Urumqi, Almaty, and Tashkent serving as hubs. Structural entropy shows that highway connections are balanced, aviation links are concentrated around core hubs, and the integrated network reflects the coexistence of core-hub agglomeration and multimodal coverage expansion. Modal diversity entropy indicates that high connectivity does not necessarily imply balanced modal configuration, and 41.8% of nodes remain dependent on a single mode. Community detection reveals local cohesion and global segmentation, while accessibility analysis identifies a Western China core and Central Asian periphery. Sensitivity analyses based on common-node normalization, travel-time weighting, and alternative modal weights confirm the robustness of the findings. These results provide an integrated diagnostic framework for identifying structural concentration, modal imbalance, and accessibility inequality in cross-border multimodal transportation networks.

## 1. Introduction

With the continued advancement of the Belt and Road Initiative, connectivity between China and the five Central Asian countries has moved toward a stage of higher-quality development. Transportation infrastructure is fundamental to physical connectivity because it supports factor flows, economic and trade cooperation [1,2] and industrial coordination [3]. The continued implementation of cross-border transportation projects, such as the China–Europe Railway Express and the China–Kyrgyzstan–Uzbekistan Railway, has helped establish efficient land corridors between Western China and Central Asia [4]. Western China also serves as a major consolidation area for China–Europe Railway Express services and an aviation gateway to Central Asia. Its multilayer transportation network, composed of railways, aviation routes, and highways, plays an important role in connecting China’s eastern hinterland with passenger and freight flows to Central Asia [5,6]. However, the improvement of transportation infrastructure does not necessarily mean that cross-border transportation networks can achieve efficient coordination [7]. Influenced by corridor layout, border-port connectivity, and multimodal transfer, connectivity between China and Central Asia still faces several shortcomings, which constrain the efficiency of regional factor flows and the improvement of economic and trade cooperation [8,9]. In actual operations, key hubs such as Xi’an, Urumqi, and Almaty still lack clearly defined functional hierarchies and efficient transport coordination mechanisms. As a result, China–Europe Railway Express services face poor coordination in border handover, train marshalling, and dispatching, which affects the timeliness and reliability of end-to-end transportation [10,11,12]. Meanwhile, transfer facilities and information-sharing mechanisms among railways, highways, and aviation at border ports and hub nodes remain underdeveloped. Together with terrain barriers, border management constraints, and uneven infrastructure distribution, these factors have led to pronounced spatial imbalances in the cross-border transportation network and have restricted the overall operational efficiency of the corridor [13,14,15,16].

A substantial body of research has examined Belt and Road cross-border transportation networks, transport connectivity, and the organization of multilayer transportation networks. Existing studies have shown that transport connectivity positively promotes trade [17] and value chains [18,19], while projects such as the China–Europe Railway Express have generated significant spillover effects for cities in Western China [3,20,21]. Previous research has also identified the central role of hubs such as Xi’an and Urumqi [22]. Methodologically, complex network analysis has been widely applied to studies of railway, highway, aviation, and integrated transportation networks. Indicators such as node degree, betweenness centrality, clustering coefficient, and modularity can effectively identify key nodes, network hierarchies, and community organization structures [23,24,25,26,27]. Studies of multilayer and multimodal transportation networks further emphasize that different transportation modes are not simply arranged in parallel. Instead, they form interlayer complementarity and functional coupling through hub nodes and transfer facilities [28,29,30,31,32,33,34,35]. Accessibility models are also widely used to evaluate the spatial service capacity of transportation networks and regional differences in connectivity, providing an important tool for assessing the functional performance of transportation networks [33,36,37]. However, traditional complex network indicators have difficulty capturing the balance of connection distribution and differences in multimodal configuration. Information entropy can characterize the equilibrium of system element distributions and thus provides a complementary perspective for network structure analysis [38,39]. Among entropy-based measures, structural entropy is used to evaluate the overall distribution of network connections, whereas modal diversity entropy reflects the degree of balance in a node’s configuration across rail, highway, and aviation transportation [40].

In summary, three gaps remain in the existing literature. First, most studies focus on a single transportation mode or domestic transportation networks, while insufficient attention has been paid to the Western China–Central Asia cross-border multimodal transportation network jointly formed by railways, highways, and aviation routes [32]. Second, although traditional topological indicators can identify key nodes and network hierarchies, they provide limited explanations of the balance of connection distribution, dependence on core hubs, and differences in modal configuration across nodes. Third, an integrated analytical framework linking structural characteristics, community organization, and accessibility has not yet been fully developed. Existing studies mostly examine pairwise relationships among these dimensions [41,42,43,44], and few have explained the structural heterogeneity and multimodal coordination weaknesses of cross-border transportation networks from an entropy perspective.

To address these gaps, this study focuses on the western provinces of China and the five Central Asian countries. It constructs railway, highway, aviation, and integrated transportation networks and applies complex network theory [45], community detection algorithms [46], accessibility models [47], structural entropy [39], and modal diversity entropy [40] to reveal the structural, organizational, and functional characteristics of the cross-border transportation network. It should be noted that this study does not propose a new Shannon entropy formula. Rather, its contribution lies in adapting and integrating established entropy-based indicators into a cross-border multimodal transportation network framework. Specifically, the study extends prior applications in three ways: first, by comparing single-mode and integrated transportation networks under a unified 376-city node setting; second, by combining structural entropy and modal diversity entropy with community detection and accessibility analysis within a structure–organization–function framework; and third, by introducing common-node and travel-time-weighted sensitivity analyses to examine the robustness and interpretation boundaries of the entropy-based results.

## 2. Materials and Methods

### 2.1. Materials

This study is based on data reflecting conditions in 2024. The research area covers the western provinces of China and the five Central Asian countries, with cities at different administrative levels within the region selected as the basic nodes of the transportation network. The analysis focuses on the physical network data of three transportation modes: railways, highways, and aviation routes.

The data used in this study come from four main sources. First, vector map data were obtained from the National Geomatics Center of China and the Tianditu platform to define the study area and administrative boundaries. Second, transportation network data were acquired from OpenStreetMap to identify the physical connections of railway, highway, and aviation routes within the study area. Third, spatial coordinate data of nodes were collected from the APIs of Baidu Maps, Amap, and Google Maps. Fourth, travel time data were obtained from the China Railway 12306 platform and Amap.

### 2.2. Research Methods

This study applies complex network analysis and accessibility assessment to examine single-mode and integrated transportation networks from three dimensions: topological structure, community organization, and accessibility performance.

#### 2.2.1. Accessibility

Accessibility originates from classical location theory and was proposed by Hansen [48]. It refers to the degree of interaction opportunities among nodes in a transportation network. In this study, the accessibility of node *i*, denoted as *A_i_^m^*, is measured by the sum of the reciprocals of travel times from node *i* to all other nodes in the network. The shorter the travel time, the higher the accessibility of the node. The calculation formula is as follows:

(1) $$A_{i}^{m}=\sum_{j\neq i}\frac{1}{t_{ij}^{m}}$$

where *A_i_^m^* denotes the accessibility of node *i* under transportation mode *m*, *t_ij_^m^* denotes the travel time from node *i* to node *j* under mode *m*, and *m* ∈ {R,H,A} represents railway, highway, and aviation, respectively. If node *j* is not reachable from node *i* under mode *m*, the corresponding term is treated as zero.

To comprehensively evaluate the overall accessibility performance of nodes under the allocation of multiple transportation resources, this study constructs a weighted accessibility model for the integrated transportation network.

Previous studies have used different weighting strategies in integrated transportation accessibility analysis. Some studies constructed intermodal networks without assigning modal weights, whereas others introduced mode-specific weights according to regional transport conditions [49,50]. The 2024 Statistical Bulletin on the Development of the Transportation Industry indicates that highways remain dominant in China’s overall transport system, while railways and aviation play different supplementary roles [51]. Considering both this general transport structure and the functional characteristics of medium- and long-distance cross-border corridors, this study assigns weights of 0.45, 0.40, and 0.15 to railways, highways, and aviation, respectively. Railways serve as the backbone mode for trunk transportation, highways provide extensive collection, distribution, and short-distance connection functions, and aviation supports time-sensitive passenger transport and high-value-added freight transport. Integrated transportation accessibility is then obtained by weighted aggregation of the accessibility values of the three single-mode networks. The calculation formula is as follows:

(2) $$A_{i}^{I}=\sum_{m\in M}w_{m}A_{i}^{m}$$

where *A_i_^I^* denotes the integrated transportation accessibility of node *i*, *M* = {R,H,A}, and *w_m_* denotes the weight assigned to mode *m*. In this study, *w_R_* = 0.45, *w_H_* = 0.40, and *w_A_* = 0.15.

This superimposed effect reflects the strategic position of a node as a regional integrated hub. The calculation results can identify hubs that achieve significant improvements in accessibility through the integration of transportation modes, as well as low-accessibility areas caused by poor intermodal connections or the absence of certain transportation modes.

To examine whether the integrated accessibility results are sensitive to the assignment of modal weights, this study conducted a scenario-based sensitivity analysis. The baseline scenario follows the original setting of rail = 0.45, road = 0.40, and air = 0.15. An equal-weight scenario was first introduced as a neutral benchmark. In addition, one-at-a-time perturbation scenarios were designed by increasing and decreasing the weight of each transportation mode by 20%, respectively, while proportionally redistributing the remaining weight between the other two modes. Spearman rank correlation coefficients between the baseline scenario and each alternative scenario were then calculated to evaluate the overall stability of node rankings. The overlap ratio of the top 10 nodes was also used to examine whether the main high-accessibility hubs remained stable under different weighting assumptions.

#### 2.2.2. Complex Network

Using the L-space modeling approach, this study defines cities as network nodes and the actual routes of various transportation modes as network edges, thereby constructing physical network models for the railway, highway, aviation and integrated transportation networks. Three indicators—degree distribution, betweenness centrality, and clustering coefficient—are used to quantify network topology, identify key nodes, and characterize the hierarchical structure of the network. The calculation formulas and connotations of each indicator are as follows:

(1) Degree

(3) $$k_{i}=\sum_{j=1}^{N}a_{ij}$$

where *k_i_* denotes the degree of node *i*, *N* denotes the total number of nodes in the network, and *a_ij_* is the element of the adjacency matrix. If nodes *i* and *j* are directly connected, *a_ij_* = 1; otherwise, *a_ij_* = 0. The degree distribution is then obtained from the frequency distribution of *k_i_* across all nodes and is used to describe the overall heterogeneity of network connections.

(2) Betweenness centrality

(4) $$C_{B}(i)=\sum_{s\neq i\neq t}\frac{\sigma_{st}(i)}{\sigma_{st}}$$

where *C_B_(i)* denotes the betweenness centrality of node *i*. Nodes *s* and *t* represent any two nodes in the network other than node *i*. *σ_st_* denotes the total number of shortest paths between nodes *s* and *t*, while *σ_st_(i)* denotes the number of those shortest paths that pass through node *i*. The ratio *σ_st_(i)*/*σ_st_* represents the share of shortest paths between nodes *s* and *t* that depend on node *i*. A higher *C_B_(i)* indicates a stronger intermediary or transfer role in the transportation network.

(3) Clustering coefficients

(5) $$C_{i}=\frac{2e_{i}}{k_{i}(k_{i}-1)}$$

where *C_i_* denotes the clustering coefficient of node *i*, *k_i_* denotes the degree of node *i*, and *e_i_* denotes the actual number of edges among the neighbors of node *i*. The denominator *k_i_*(*k_i_* − 1)/2 represents the maximum possible number of edges among these neighbors. When *k_i_* < 2, *C_i_* is set to 0. A higher clustering coefficient indicates stronger local connectivity and greater local redundancy.

#### 2.2.3. Community Detection

This study uses the Louvain community detection algorithm in Gephi 0.10.1 [46] to identify the community structures of the railway, highway, aviation, and integrated transportation networks. Based on the principle of modularity optimization, this algorithm can identify groups of nodes that are relatively densely connected and frequently linked within the network. Modularity is used to measure the significance of community partitioning results. A higher modularity value indicates a more pronounced community structure, stronger intra-community connections, and more distinct separation between communities.

The Louvain community detection algorithm was implemented in Gephi. The baseline analysis used the default resolution parameter of 1.0. To examine the sensitivity of community partitions to the resolution parameter, this study further tested six resolution values: 0.5, 0.8, 1.0, 1.2, 1.5, and 2.0. These values were selected to cover community partitions below and above the default setting. In all runs, the Randomize and Use edge weights options were enabled. Since all edges were assigned a unit value in the standard Weight field, the community detection was essentially based on the unweighted topological structure of the networks. For the integrated transportation network, the multilayer representation with highway-layer nodes, railway-layer nodes, airport nodes, and intermodal transfer links was retained.

#### 2.2.4. Measurement of Entropy Indicators

To further characterize the structural balance of the cross-border transportation network and the differences in multimodal transportation configuration, this study adopts two established entropy-based indicators, namely structural entropy and modal diversity entropy, on the basis of traditional complex network indicators. Structural entropy is derived from Shannon entropy and is used here to measure the degree of balance in the distribution of node connections across different transportation networks. Modal diversity entropy follows the logic of modal composition entropy in previous transport network studies and is used here to measure the degree of balance in the configuration of an individual node across three transportation modes: railways, highways, and aviation routes. The methodological contribution of this study does not lie in proposing new entropy formulas, but in adapting these indicators to a cross-border multimodal transportation network and combining them with common-node normalization, travel-time-weighted sensitivity analysis, community detection, and accessibility assessment.

To avoid repeating equivalent entropy formulas under different analytical settings, this study defines two index sets. Let *M* = {R, H, A} denote the three transportation modes, namely railway, highway, and aviation, and let *G* = *M* ∪ {I} denote the set consisting of the three single-mode networks and the integrated transportation network. This study uses *x_i_^g^* as a unified input variable for entropy calculation, where *x_i_^g^* denotes the connection intensity of node *i* in network *g*. In the degree-based analysis, *x_i_^g^* = *k_i_^g^*, where *k_i_^g^* is the degree of node *i* in network *g*. For the integrated transportation network, the input variable is defined as *x_i_^I^* = ∑*_m__∈M_ x_i_^m^*. In the travel-time-weighted sensitivity analysis, *x_i_^m^* = *s_i_^m^*, where *s_i_^m^* is the travel-time-weighted strength of node *i* under transportation mode *m*. Accordingly, the weighted input of the integrated network is defined as *x_i_^I^* = ∑*_m__∈M_ s_i_^m^*.

(1) Structural Entropy

Structural entropy is used to describe the degree of balance in the distribution of network connections among nodes. Based on the unified input variable *x_i_^g^*, the connection proportion, structural entropy, and normalized structural entropy are calculated as follows:

(6) $$p_{i}^{g}=\frac{x_{i}^{g}}{\sum_{r=1}^{N}x_{r}^{g}},\;H_{s}^{g}=-\sum_{i=1}^{N}p_{i}^{g}\ln p_{i}^{g},\;H_{s,\mathrm{norm}}^{g}=\frac{H_{s}^{g}}{\ln N}$$

where *g* ∈ *G* denotes the network type, including the railway, highway, aviation, and integrated transportation networks; *N* denotes the number of nodes included in the corresponding network; *x_i_^g^* denotes the connection intensity of node *i* in network *g*; *p_i_^g^* denotes the share of node *i*’s connection intensity in the total connection intensity of network *g*; *H_s_^g^* denotes the structural entropy of network *g*; and *H_s,norm_^g^* denotes normalized structural entropy. A higher value of *H_s,norm_^g^* indicates a more even distribution of network connections, whereas a lower value indicates stronger concentration around a limited number of hub nodes. When *p_i_^g^* = 0, the corresponding entropy term is treated as zero.

To address the potential influence of network size on the comparison of structural entropy across different modal networks, this study further conducted a common-node sensitivity analysis. In this analysis, the same structural entropy formulation was recalculated using a unified set of 376 city-level nodes. For cities not covered by a given transportation mode, the corresponding *x_i_^g^* was set to zero. The normalized structural entropy was then calculated with *N* = 376. This treatment enables the railway, highway, aviation, and integrated transportation networks to be compared under the same node scale, thereby reducing the influence of different network sizes and coverage ranges on cross-network structural entropy comparison. It should be noted that this indicator remains a topological entropy measure. It mainly reflects the balance of connection distribution and does not directly represent actual transport capacity, service frequency, or passenger and freight flows.

(2) Modal Diversity Entropy

Modal diversity entropy is used to measure the degree of balance in a node’s connections across railway, highway, and aviation. Based on the unified input variable *x_i_^m^*, the modal connection proportion, modal diversity entropy, and normalized modal diversity entropy are calculated as follows:

(7) $$q_{i}^{m}=\frac{x_{i}^{m}}{\sum_{r\in M}x_{i}^{r}},\;H_{d,i}=-\sum_{m\in M}q_{i}^{m}\ln q_{i}^{m},\;H_{d,i,\mathrm{norm}}=\frac{H_{d,i}}{\ln|M|}$$

where *q_i_^m^* denotes the proportion of node *i*’s connection intensity assigned to transportation mode *m*; *H_d,i_* denotes the modal diversity entropy of node *i*; *H_d,i,norm_* denotes normalized modal diversity entropy; and ∣*M*∣ denotes the number of transportation modes. In this study, *M* = {R,H,A} and ∣*M*∣= 3. When *q_i_^m^* = 0, the corresponding entropy term *q_i_^m^*ln(*q_i_^m^*) is treated as zero. A higher value of *H_d,i,norm_* indicates a more balanced multimodal configuration, whereas a lower value indicates greater dependence on a single mode of transportation.

Because structural entropy and modal diversity entropy are calculated from node degrees in the main analysis, they mainly measure the topological balance of connection distribution but cannot fully represent transport capacity, service frequency, passenger and freight flows, or operational efficiency. Therefore, a travel-time-weighted entropy sensitivity analysis was conducted to examine the robustness of the entropy-based conclusions. Due to the lack of complete and comparable passenger flow, freight flow and service frequency data for all cross-border transportation links, inverse travel time was used as a consistent functional weight. Duplicate undirected city pairs were aggregated by retaining the minimum travel time, and self-loops generated by city-level node aggregation were excluded. The travel-time-weighted strength of node *i* under transportation mode *m* was calculated as follows:

(8) $$s_{i}^{m}=\sum_{j\neq i}\frac{a_{ij}^{m}}{t_{ij}^{m}}$$

where *s_i_^m^* denotes the travel-time-weighted strength of node *i* under transportation mode *m*; *a_ij_^m^* indicates whether nodes *i* and *j* are connected under mode *m*, and *t_ij_^m^* denotes the travel time between nodes *i* and *j* under mode *m*. If nodes *i* and *j* are connected, *a_ij_^m^* = 1; otherwise, *a_ij_^m^* = 0 and the corresponding term is treated as zero. The weighted structural entropy and weighted modal diversity entropy were calculated by setting *x_i_^m^* = *s_i_^m^* in the entropy formulas above and were then compared with the degree-based results.

#### 2.2.5. Analytical Framework and Research Procedure

**Figure 1:**
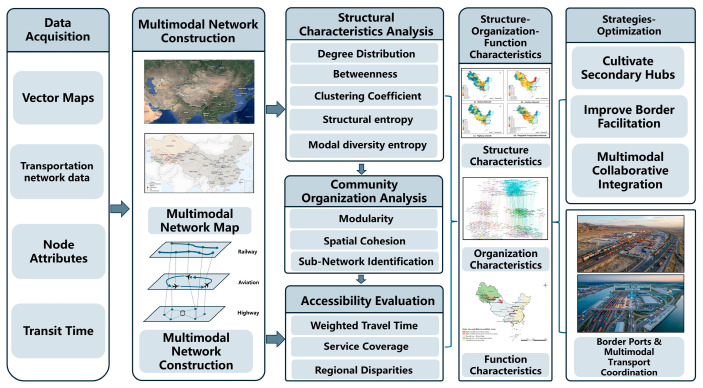
Research Framework. Arrows indicate the sequence of the analytical workflow, and the boxes represent the main research stages and analytical modules.

The overall analytical framework and research procedure are illustrated in Figure 1. Based on the construction of railway, highway, aviation, and integrated transportation networks, this study analyzes the Western China–Central Asia cross-border multimodal transportation network from the perspectives of topological structure, structural entropy, modal diversity entropy, community organization, and accessibility differentiation, and subsequently proposes targeted optimization strategies.

## 3. Results

### 3.1. Structural Characteristics and Entropy Analysis of the Western China–Central Asia Cross-Border Transportation Network

**Figure 2:**
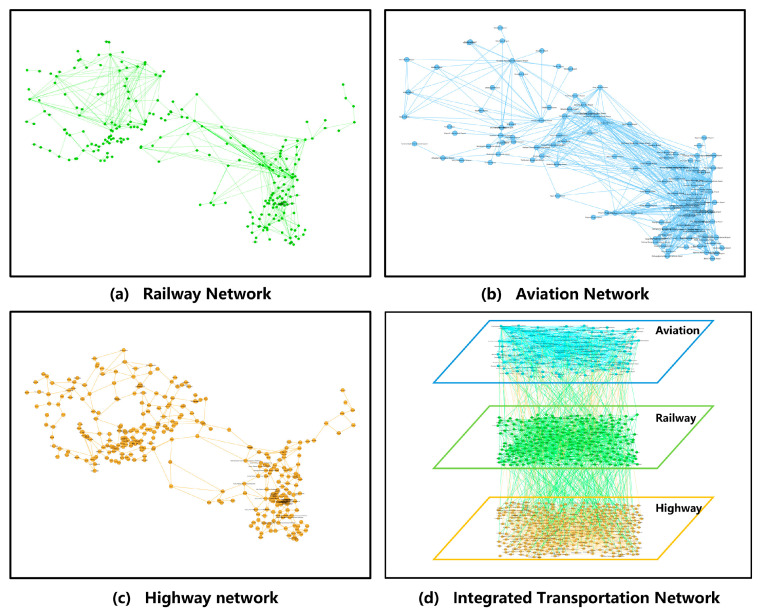
Construction of the Western China–Central Asia Cross–Border Transportation Network.

**Figure 3:**
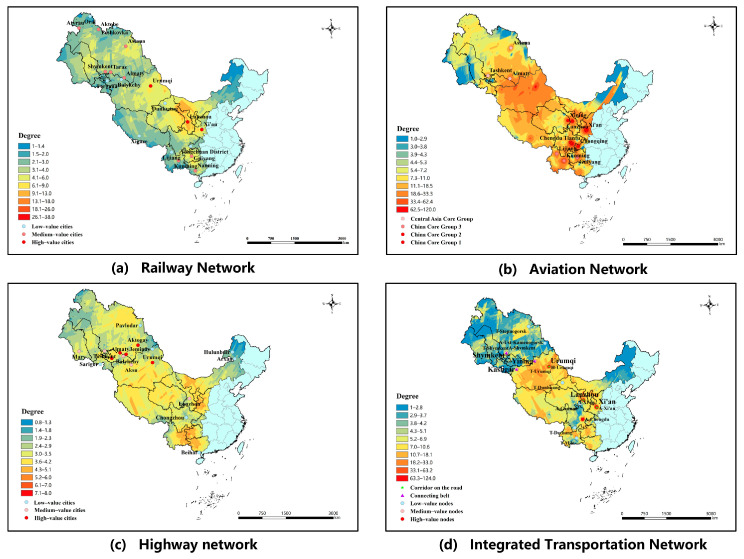
Node Degree and Spatial Pattern of the Transportation Network.

**Figure 4:**
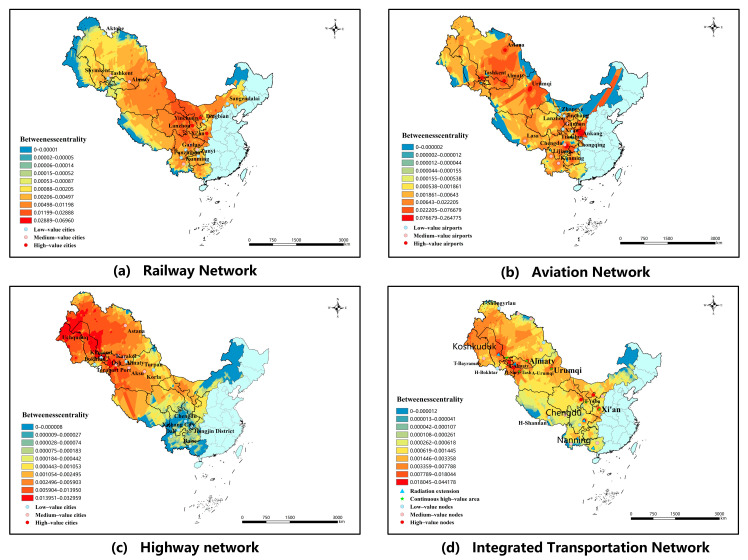
Betweenness Centrality of the Transportation Network.

**Figure 5:**
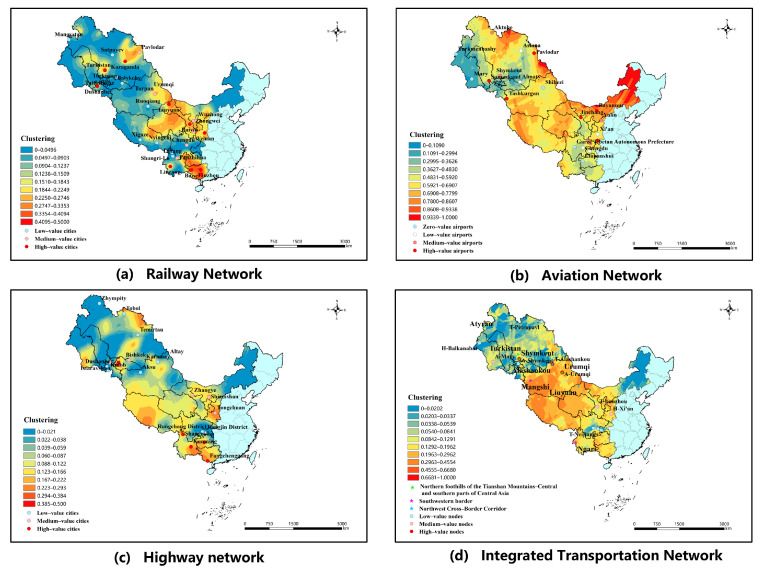
Clustering Coefficient of the Transportation Network.

This study constructs the transportation networks between Western China and the five Central Asian countries and analyzes their characteristics. Figure 2, Figure 3, Figure 4 and Figure 5 each contain four subfigures, corresponding to the railway network, aviation network, highway network, and integrated transportation network, respectively.

#### 3.1.1. Construction of the Cross-Border Transportation Network

To reveal the multilayered architecture of the Western China–Central Asia cross-border transportation network, this study constructs railway, aviation, highway, and integrated transportation networks, whose spatial patterns are shown in Figure 2. Different transportation modes vary significantly in their topological forms and functional positioning. The railway network exhibits a “core–periphery” structure and extends linearly along major land corridors, with core connections concentrated in hub areas of Western China and eastern Central Asia, while peripheral areas are relatively sparsely connected. The aviation network shows a typical “hub-and-spoke” pattern, with its core concentrated in a few central nodes characterized by high time efficiency and broad coverage. The highway network has the widest coverage: it forms a mesh-like pattern in Western China and the core areas of Central Asia, while cross-border areas and regions constrained by geographical barriers are mostly organized as linear corridors, making it an important foundation for regional distribution and last-mile connections. The integrated transportation network reflects multimodal complementarity and forms a composite transportation system composed of rapid aviation connections, railway backbone support, and highway-based terminal coverage. This system can, to some extent, compensate for the time-efficiency or spatial constraints of single transportation modes and provide integrated support for regional passenger mobility and freight transport.

#### 3.1.2. Topological Characteristics Analysis of the Cross-Border Transportation Network

(1) Node Degree and Spatial Pattern

As shown in Figure 3, the degree distribution reveals the connection breadth of nodes in the cross-border transportation network and shows different organizational patterns across transportation modes. The railway network follows a corridor-oriented structure along major trunk lines such as the Longhai–Lanzhou–Xinjiang corridor. High-degree nodes such as Xi’an, Lanzhou, and Urumqi form the main framework for cross-regional railway connections, while peripheral areas remain weakly covered. The aviation network shows a polarized hub-and-spoke structure, with connections concentrated around major hubs such as Xi’an, Urumqi, and Almaty. The highway network has the broadest spatial coverage and forms a more extensive mesh-like structure in the core areas of Western China and Central Asia. The integrated transportation network combines the corridor support of railways, the hub radiation of aviation, and the regional coverage of highways, forming a composite cross-border corridor along the Xi’an–Lanzhou–Urumqi–Shymkent axis.

(2) Betweenness Centrality

As shown in Figure 4, the betweenness centrality results show clear differences in network control patterns across transportation modes. In the railway network, nodes such as Xi’an and Yinchuan play important intermediary roles because of their strong passenger and freight collection and transfer functions. However, railway control capacity remains uneven, with stronger connectivity in the hinterland and weaker connectivity in frontier and cross-border areas. In the aviation network, betweenness centrality is highly concentrated in a few core hubs, including Xi’an, Chengdu, and Almaty, which improves long-distance connectivity but also increases dependence on hub airports. In the highway network, intermediary functions are strongly constrained by terrain and corridor layout, with nodes such as Osh and Uchkuduk acting as key bottlenecks. In the integrated network, high-betweenness nodes are mainly distributed along the Xi’an–Lanzhou–Urumqi-Kashgar–Tashkent–Almaty axis, indicating that multimodal complementarity further strengthens the strategic role of core corridor nodes.

(3) Clustering Coefficient

The clustering coefficient measures the density of local connections around a node and reflects local redundancy and structural resilience. As shown in Figure 5, the railway network forms several high-clustering areas in Southwest China, Northwest China, and Central Asian industrial regions, but many nodes remain locally weakly connected. The aviation network shows a negative relationship between hub importance and local clustering: large hubs such as Xi’an and Chengdu have relatively low clustering coefficients because of their radial connections, whereas some regional airports show higher local clustering. The highway network exhibits strong spatial differentiation. Some cities, such as Dushanbe and Tashkent, are organized mainly through linear or radial road structures and therefore have limited local redundancy. In the integrated transportation network, high-clustering nodes are concentrated around cross-border gateways such as Alashankou and regional hubs such as Urumqi and Shymkent. Through multimodal connections, the integrated network reduces the local isolation observed in single-mode networks and strengthens regional transfer capacity.

The topological analysis reveals a cross-border transportation network characterized by a prominent core, weak periphery, and strong dependence on key gateway nodes. Xi’an, Urumqi, Tashkent, and Almaty have high degree and betweenness centrality and therefore serve as core hubs for cross-regional transport flows, while gateway nodes such as Alashankou, Khorgos, and Osh control key cross-border corridors despite their relatively limited connectivity. This contrast between core concentration and peripheral weakness directly influences community formation: high-centrality nodes tend to form functional core communities, whereas structurally weak corridor segments often become community boundaries. The following section applies the Louvain algorithm to identify community structures and uses modularity to evaluate the strength of community partitioning.

#### 3.1.3. Structural Entropy and Modal Diversity Entropy Analysis of the Cross-Border Transportation Network

Based on traditional complex network indicators, such as degree distribution, betweenness centrality, and clustering coefficient, this study further applies structural entropy and modal diversity entropy as complementary diagnostic indicators to reveal the degree of balance in the distribution of connections within the cross-border transportation network and the multimodal transportation configuration characteristics of different nodes. Traditional centrality indicators are mainly used to identify key nodes, whereas entropy indicators can further explain the heterogeneity of network structure from the perspectives of overall distribution and multimodal balance.

As shown in Table 1, there are clear differences in structural entropy among the networks of different transportation modes. Under the original network-specific setting, the highway network has the highest normalized structural entropy, indicating that highway connections are relatively evenly distributed among the nodes covered by the highway network. The railway network also shows a generally balanced internal connection distribution, although its corridor-oriented structure still produces some trunk-line agglomeration. The aviation network has a lower normalized structural entropy, reflecting stronger concentration around a limited number of core airport cities and a typical hub-and-spoke structure. The integrated transportation network has an original normalized structural entropy of 0.8718. This relatively low value suggests that, within the integrated network, core cities still concentrate on strong comprehensive connection advantages after multimodal superposition. However, because the railway, highway, aviation, and integrated transportation networks differ in node size and spatial coverage, the cross-network comparison of normalized structural entropy should be interpreted with caution. To reduce the influence of different network sizes, this study further recalculated structural entropy under a unified set of 376 city-level nodes. For cities not covered by a given transport mode, the corresponding degree was set to zero, and all networks were normalized by ln (376).

Because normalized structural entropy ranges from 0 to 1, values closer to 1 indicate a more even distribution of connections, whereas lower values indicate stronger concentration around a limited number of nodes. However, there is no universally fixed threshold for defining “high” or “low” structural entropy in multimodal transportation networks, because entropy values are affected by network boundary, node definition, edge representation, and normalization method. Therefore, the entropy values in this study are interpreted mainly through three benchmarks: the theoretical normalized scale, the relative comparison among railway, highway, aviation, and integrated networks under the same setting, and the travel-time-weighted sensitivity analysis.

Using this relative interpretation framework, the common-node results show that the highway network still has the highest normalized structural entropy, with a value of 0.9529, indicating the most balanced regional coverage and connection distribution across the study area. In contrast, the aviation network has the lowest common-node normalized structural entropy, with a value of 0.7074, reflecting the strong concentration of aviation connections around a limited number of core airport cities. The common-node normalized structural entropy of the railway network is 0.8563, indicating that the spatial selectivity of railway coverage becomes more evident when cities without railway connections are also included in the unified node set. The integrated transportation network has a common-node normalized structural entropy of 0.8718, which is higher than those of the railway and aviation networks but lower than that of the highway network. This result suggests that multimodal superposition does not simply lead to a unidirectional strengthening of connection concentration. Rather, the integrated transportation network reflects the coexistence of core-hub agglomeration and multimodal coverage expansion. Therefore, the structural entropy results should be understood as degree-based topological evidence of connection distribution, rather than direct evidence of actual transport capacity, service frequency, or traffic volume.

**Figure 6:**
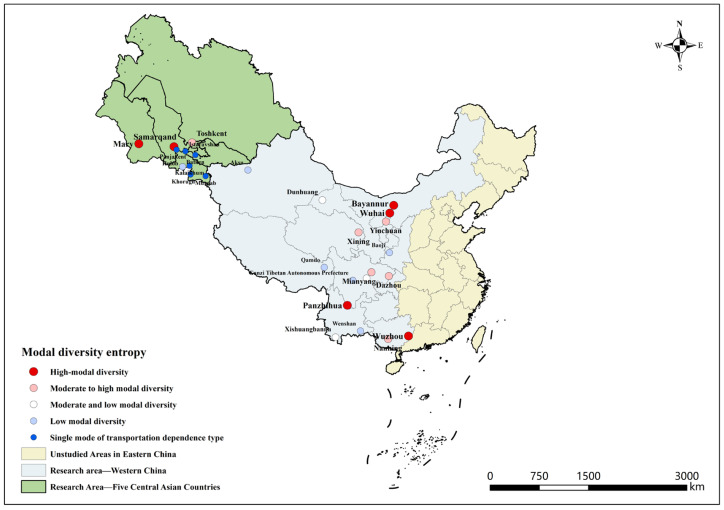
Spatial Distribution of Node Modal Diversity Entropy in the Western China–Central Asia Cross-Border Integrated Transportation Network.

To reveal the degree of configurational balance among railway, highway, and aviation transportation modes across different nodes, this study calculates the modal diversity entropy of 376 city-level nodes in the integrated transportation network. A higher modal diversity entropy indicates a more balanced distribution of a node’s connections among the three transportation modes and stronger topological multimodal coordination capacity. Conversely, a lower value indicates a higher dependence on a particular transportation mode and a more homogeneous modal structure. It should be noted that the modal diversity entropy used in this study is also degree-based. Therefore, it reflects the balance of modal configuration from a topological connection perspective, rather than actual transport capacity, service frequency, or passenger and freight flows. Figure 6 shows the spatial distribution of node modal diversity entropy in the integrated transportation network.

The results in Table 2 show that overall connectivity is not necessarily consistent with modal balance. Highly connected nodes do not always have high modal diversity entropy, whereas some medium-sized nodes show stronger multimodal coordination potential because their railway, highway, and aviation connections are more evenly distributed. Wuhai, Wuzhou, and Samarkand have normalized modal entropy values close to 1, indicating relatively balanced multimodal configurations. In contrast, nodes with medium–low modal coordination are mostly characterized by the absence or weakness of a particular transportation mode. Chengdu, Dunhuang, and Xishuangbanna show clear aviation-dominated structures, while Qal’ai Khumb, Khorog, and Murghab are typical single-mode dependence nodes. These results suggest that modal diversity entropy can identify node-level modal weaknesses that cannot be captured by total degree alone.

From the full-sample statistics, single-mode dependence nodes account for 41.8% of the 376 city-level nodes, indicating that a considerable proportion of nodes in the Western China–Central Asia cross-border integrated transportation network still rely on a single transportation mode, with insufficient overall transport redundancy and substitutability. Therefore, in optimizing the cross-border transportation network, hub value should not be assessed solely on the basis of node centrality or total degree. Instead, modal diversity entropy should also be incorporated to identify modal weaknesses at individual nodes.

To further examine whether the entropy results are sensitive to the degree-based measurement, this study recalculated structural entropy and modal diversity entropy using inverse travel time as the connection weight. The comparison between degree-based and travel-time-weighted entropy indicators is shown in Table 3.

The weighted results are generally consistent with the degree-based entropy analysis. The highway network still has the highest weighted structural entropy, whereas the aviation network remains the most concentrated single-mode network. The weighted structural entropy of the integrated transportation network is 0.9003, indicating that functional connections are more evenly distributed when travel-time efficiency is considered.

For modal diversity entropy, the mean normalized value changes only slightly from 0.3849 to 0.3812, and the share of single-mode dependence nodes remains 41.8%. The share of strongly imbalanced nodes increases slightly from 45.5% to 47.9%, suggesting that modal imbalance remains evident after travel-time efficiency is considered. These results indicate that the main entropy-based conclusions are not solely an artifact of using node degree. Nevertheless, the differences between degree-based and weighted results also show that connection quantity and functional transport efficiency are not fully equivalent.

### 3.2. Community Organization Characteristics of the Western China–Central Asia Cross-Border Transportation Network

The agglomeration effect of core nodes and the connectivity deficiencies of peripheral areas revealed by the network topology provide an important basis for the formation of community organization. This section focuses on the characteristics of community organization itself. By identifying the community composition of each transportation network, it clarifies the position of high-centrality nodes within communities and the relationship between structurally weak areas and community boundaries, thereby laying a foundation for the subsequent in-depth analysis of the relationship between network structure and organization.

#### 3.2.1. Community Differences Among Single-Mode Transportation Networks

The community structures of the railway, aviation, and highway networks differ significantly, reflecting differences among transportation modes in terms of spatial constraints, corridor organization, and cross-regional connectivity. Ground transportation networks are more strongly constrained by national borders and terrain, resulting in an organizational pattern characterized by dense intra-community connections but weak inter-community links. In contrast, the aviation network is less affected by ground spatial conditions, enabling more direct cross-regional connections and a lower degree of community segmentation.

**Figure 7:**
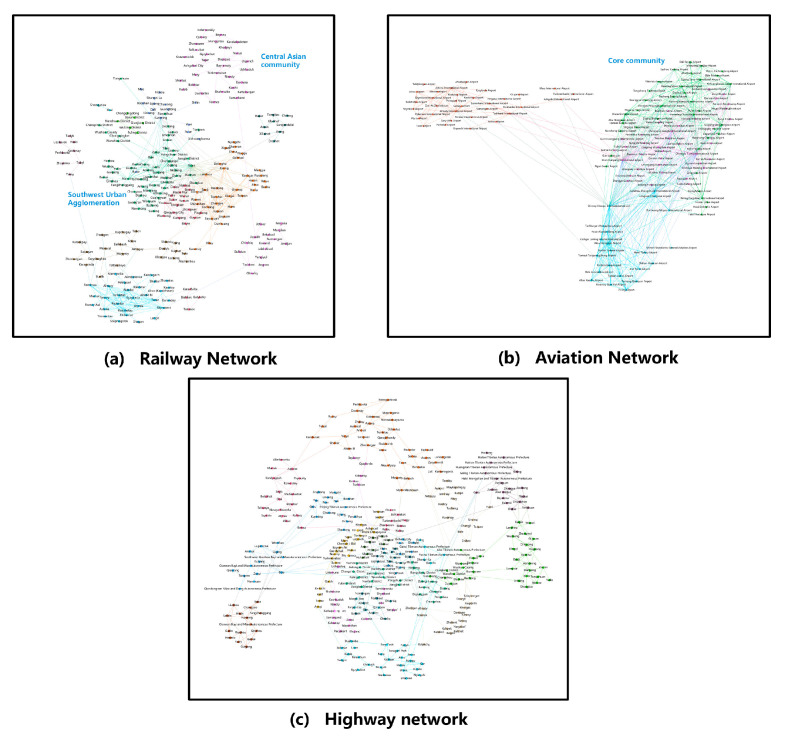
Community Structure of Single-Mode Transportation Networks between Western China and Central Asia. Node colors distinguish different communities, while the lines represent connections between nodes and do not indicate additional categories.

As shown in Figure 7, the railway network is divided into 12 communities, showing a distinct core–periphery pattern and regional characteristics. The community composed of major cities in Southwest China, such as Chengdu-Chongqing, Kunming, and Guiyang, forms an important distribution and transfer core for regional railway transportation. By contrast, the community formed by railway nodes in the five Central Asian countries shows a relatively high degree of independence, indicating that weak coupling and dependence on cross-border corridors still exist between the railway networks of Western China and Central Asia. The highway network exhibits the most pronounced community segmentation: intra-community connectivity is relatively strong, whereas cross-border and cross-regional links remain limited. Communities in high-altitude and peripheral areas still show strong isolation, suggesting that national borders, mountainous and plateau terrain, and regional infrastructure disparities impose significant constraints on the organization of the highway network. Although the aviation network also shows certain clustering characteristics, inter-community connections are relatively close and the overall level of integration is higher. The community centered on Xi’an becomes the core of the entire network due to its strong aviation distribution and transfer capacity, while other communities mainly undertake feeder connections and supplementary spatial coverage for peripheral areas. To some extent, the aviation network breaks through the spatial segmentation of ground transportation networks, but its cross-regional connections remain concentrated in a few core airports.

#### 3.2.2. Modular Organization of the Integrated Transportation Network

**Figure 8:**
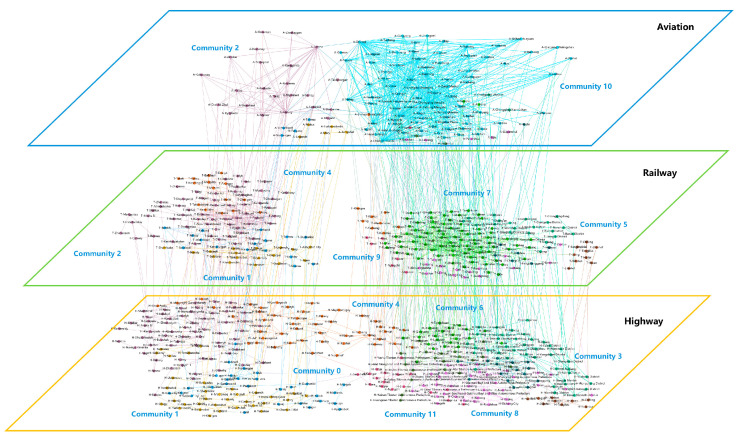
Community Structure of the Integrated Transportation Network.Node labels are based on city names, with the prefixes “A-”, “T-”, and “H-” indicating aviation-layer, railway-layer, and highway-layer nodes, respectively. The prefixes distinguish layer-specific representations of the same city, while the lines represent connections between nodes.

As shown in Figure 8, the integrated transportation network shows a clear modular structure, with a modularity value of 0.668 and 12 detected communities. Its community pattern is characterized by dominant core communities and several smaller peripheral communities. This indicates that the highway, railway, and aviation layers are not simply superimposed but are functionally connected through multimodal hubs and transfer links. In terms of community composition, several small communities, including Communities 5, 6, 8, and 11, show strong modal or geographic homogeneity and mainly serve local short-distance transport needs. In contrast, the larger integrated communities, including Communities 2, 7, and 10, show stronger heterogeneity, with multiple transportation modes and cross-regional connections combined within the same community. These large communities therefore play a more important role in long-distance and multimodal transport organization.

Community analysis indicates that the Western China–Central Asia cross-border transportation network has a clear modular structure characterized by local cohesion and global segmentation. This pattern is strongly shaped by geographical barriers, national boundaries, and uneven infrastructure development. Core communities centered on Xi’an and Almaty contrast sharply with peripheral communities. Nodes in Western China are more closely connected through relatively well-developed transportation networks, whereas many Central Asian nodes remain sparsely connected and weakly integrated. This modular segmentation and the fragility of inter-community links directly affect regional transport performance. In general, strong intra-community cohesion corresponds to higher accessibility and travel efficiency, while weakly connected inter-community areas are more likely to become low-accessibility zones. The following section further evaluates accessibility patterns across single-mode and integrated transportation networks to explain how structural and organizational characteristics shape transport efficiency.

#### 3.2.3. Robustness of Community Detection Under Alternative Resolution Parameters

To examine whether the detected community structures are sensitive to the resolution parameter of the Louvain algorithm, a resolution sensitivity analysis was conducted using six resolution values: 0.5, 0.8, 1.0, 1.2, 1.5, and 2.0. The results are shown in Table 4.

The results show that changing the resolution parameter alters the granularity of community partitioning, but the main organizational pattern remains stable. Across all resolution settings, the highway network consistently exhibits the highest modularity, ranging from 0.797 to 0.915, indicating strong local segmentation constrained by terrain, administrative boundaries, and regional road systems. The aviation network consistently shows the lowest modularity, ranging from 0.188 to 0.364, reflecting stronger cross-regional connectivity and weaker community boundaries. The integrated network has lower modularity than the highway network under all resolution settings. This result does not indicate weaker network organization; rather, it suggests that multimodal superposition introduces more cross-regional and inter-community links through railway corridors, aviation routes, and intermodal transfer edges, thereby weakening the local community boundaries observed in the highway network.

The community membership of representative hub and corridor nodes was also examined. Xi’an, Urumqi, Lanzhou, Tashkent, Almaty, and Shymkent were selected because they represent the main Chinese hubs, Central Asian hubs, and key corridor nodes identified in the topological, community, and accessibility analyses. The results show that these nodes generally remain associated with large or regionally dominant communities under most resolution settings. In the integrated multilayer network, some layer-specific splitting occurs, especially for airport nodes and gateway nodes such as Urumqi. This pattern reflects functional differentiation among highway, railway, and aviation layers rather than instability of the detected community structure. Detailed community membership results are provided in Table A1.

### 3.3. Accessibility Pattern of the Western China–Central Asia Cross-Border Transportation Network

Network topology and community structure jointly shape the spatial efficiency of regional transportation systems. Accessibility provides a functional measure for evaluating how effectively different nodes are connected within the cross-border transportation network. This section evaluates railway, aviation, highway, and integrated transportation accessibility, with a focus on cross-border spatial disparities, the radiation capacity of core hubs, and the distribution of marginal low-accessibility areas.

#### 3.3.1. Accessibility Differences Among Single-Mode Transportation Networks

(1) Railway Network Accessibility

**Figure 9:**
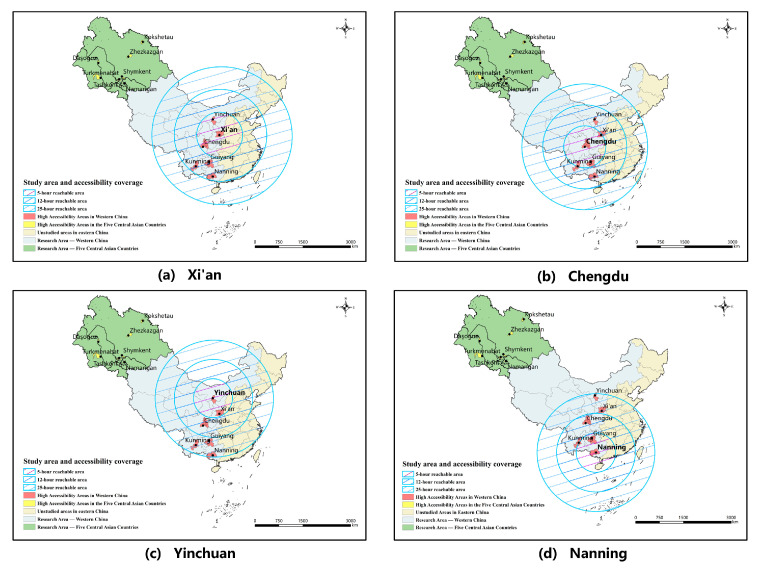
Railway accessibility in Western China.

**Figure 10:**
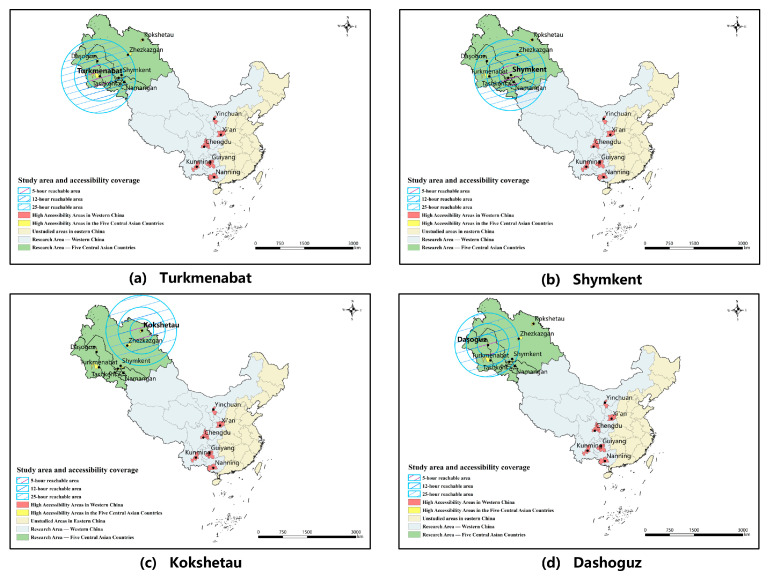
Railway accessibility in Central Asia.

As shown in Figure 9 and Figure 10, railway accessibility presents a pronounced “Western China core–Central Asian periphery” structure. Nodes in Western China generally have higher accessibility, with Xi’an, Nanning, and Yinchuan occupying dominant positions along major railway trunk lines. By contrast, Central Asian cities show lower accessibility because of weaker internal railway penetration and limited cross-border connectivity. The isochrone maps further show broader and denser railway coverage around Chinese cities such as Xi’an and Chengdu, whereas Central Asian cities such as Dashoguz and Shymkent have smaller isochrone ranges.

(2) Aviation Network Accessibility

**Figure 11:**
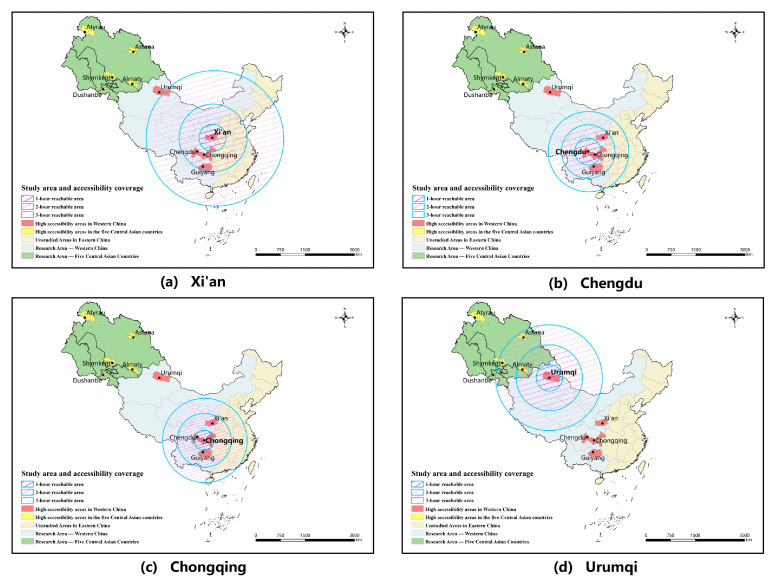
Aviation accessibility in Western China.

**Figure 12:**
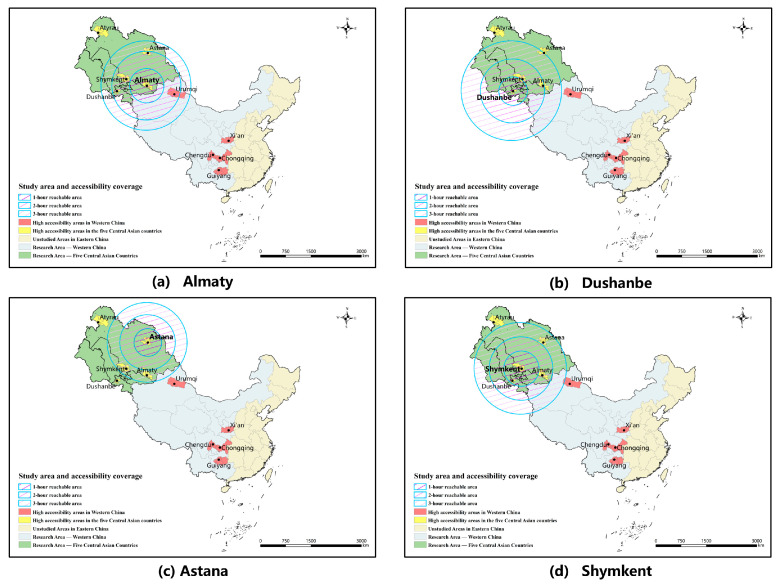
Aviation accessibility in Central Asia.

As shown in Figure 11 and Figure 12, the aviation network exhibits a strong space–time convergence effect. Aviation hubs such as Xi’an, Chengdu, Chongqing, and Urumqi occupy dominant positions and serve as key support points for the rapid distribution of regional flows and cross-border interactions. Although Central Asian cities such as Tashkent, Nur-Sultan, and Almaty also function as regional aviation nodes, their accessibility levels remain relatively lower, and their overall route density and cross-border coverage are still weaker than those of core cities in Western China. From the perspective of isochrones, Xi’an, Chengdu, and Chongqing can cover the main areas of Western China within three hours and extend toward Central Asia. Urumqi has the strongest westward radiation capacity, reaching the eastern border areas of Central Asia within three hours.

(3) Highway Network Accessibility

**Figure 13:**
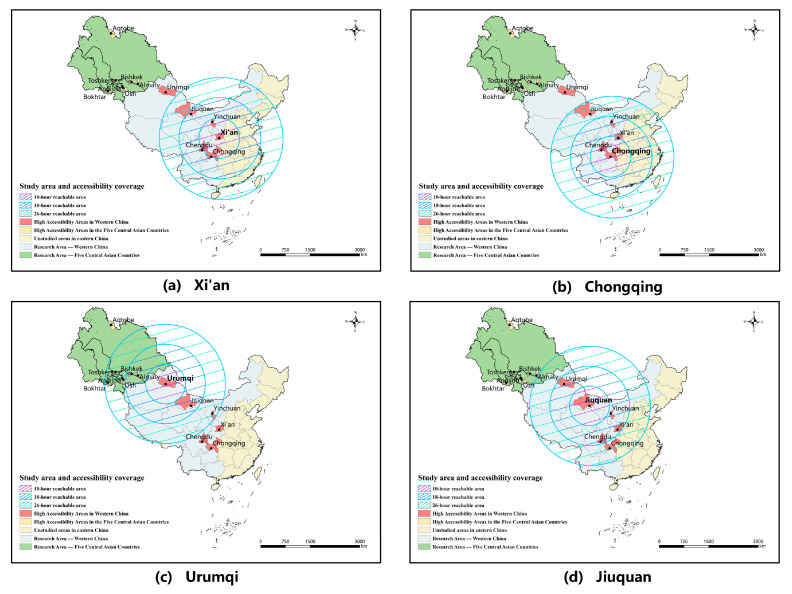
Highway accessibility in Western China.

**Figure 14:**
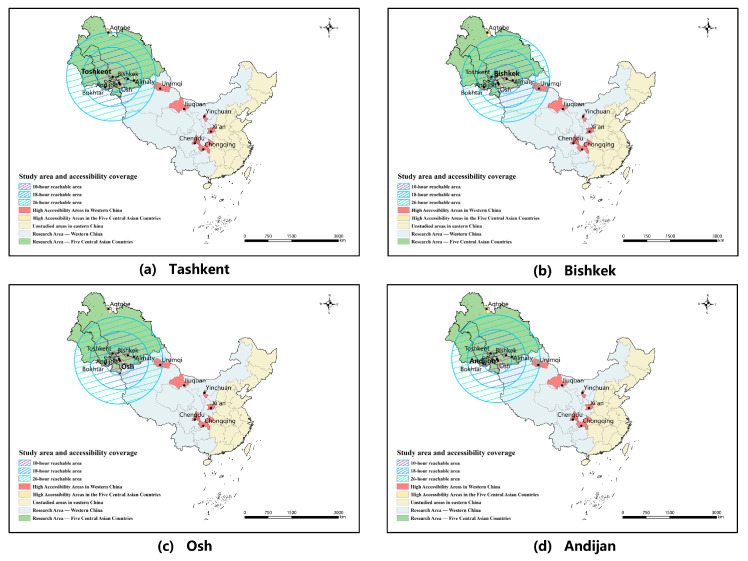
Highway accessibility in Central Asia.

As shown in Figure 13 and Figure 14, highway network accessibility shows an “agglomeration–radiation” pattern. Some cities in Western China have developed relatively strong regional accessibility based on well-established highway systems, with relatively balanced isochrone radiation. However, as westward corridors from cities such as Urumqi and Xi’an extend toward the border, accessibility declines because of lower highway grades and insufficient travel efficiency. Cities in the five Central Asian countries generally have relatively low accessibility, with clear internal polarization. A few cities, such as Andijan, remain relatively prominent because of their location near regional intersections, whereas cities located in complex mountainous environments become low-accessibility areas within the network. The highway network plays a fundamental role in regional distribution and last-mile connections, but cross-border efficiency and coverage of peripheral areas remain its main shortcomings.

All three types of networks show spatial imbalance to varying degrees. The railway network is strongly affected by corridor dependence, the aviation network relies on a few core airports, and the highway network is clearly constrained by terrain and border-crossing conditions. This indicates that a single transportation mode is insufficient to independently support the efficient operation of the Western China–Central Asia cross-border transportation network. Multimodal coordination is therefore needed to improve cross-border accessibility.

#### 3.3.2. Spatial Pattern of Integrated Transportation Accessibility

**Figure 15:**
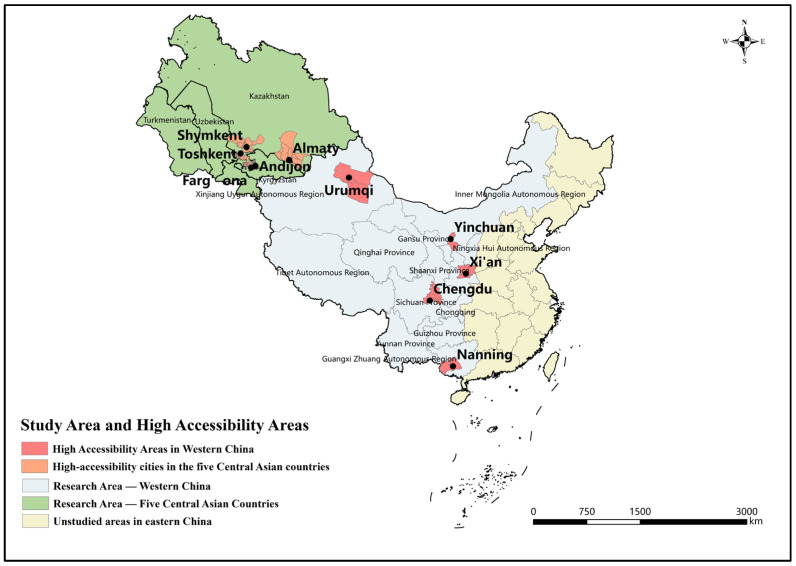
Integrated Transportation Network Accessibility.

Integrated transportation accessibility shows that multimodal superposition strengthens the advantages of core hubs. Cities such as Xi’an, Chengdu, Tashkent, and Almaty perform strongly in cross-regional connections and regional radiation, forming a major connectivity axis along Xi’an–Lanzhou–Urumqi–Shymkent in Figure 15. Compared with single-mode networks, the integrated network partly compensates for the coverage limitations of individual modes and improves the service capacity of core nodes.

However, the accessibility improvements brought by the integrated network have not spread evenly across the entire region. Some border ports, mountainous cities, and peripheral nodes in Central Asia remain at relatively low accessibility levels because of missing transportation modes, insufficient corridor connections, or weak infrastructure. Although some nodes occupy certain corridor positions, their structural advantages cannot be fully transformed into integrated accessibility advantages because their aviation, highway, or railway connections are incomplete. Combined with the modal diversity entropy results, nodes with low modal entropy and single-mode dependence are more likely to become low-accessibility areas, indicating that the balance of modal configuration at the node level has an important influence on integrated accessibility.

Overall, accessibility across different transportation networks shows a spatial pattern characterized by a Western China core and Central Asian periphery. According to the overall integrated accessibility ranking, the top-ranked nodes are mainly concentrated in Western China, while Tashkent is the only Central Asian city entering the overall top 10. To avoid confusing overall top-ranked nodes with subregional representative nodes, this study further selects representative high-accessibility nodes from Western China and Central Asia for regional comparison. The representative nodes in Western China include Xi’an, Chengdu, Nanning, Yinchuan, and Urumqi, while the representative Central Asian nodes include Tashkent, Almaty, Shymkent, Fergana, and Andijan. Although Almaty, Shymkent, Fergana, and Andijan do not enter the top 10 overall, they remain relatively high-accessibility nodes within Central Asia.

#### 3.3.3. Modal-Weight Sensitivity Analysis of Integrated Accessibility

To further test the robustness of the integrated accessibility results, eight weighting scenarios were designed, including the baseline scenario, an equal-weight scenario, and six one-at-a-time ±20% perturbation scenarios for railway, highway, and aviation weights in Table 5. The results show that the integrated accessibility rankings are highly stable under alternative weighting schemes. Table 6 shows that the Spearman rank correlation coefficients between the baseline scenario and the seven alternative scenarios range from 0.9556 to 0.9977, indicating a high degree of consistency in the overall node rankings. The top 10 overlap ratio is 8/10 under the equal-weight scenario and 9/10 under all ±20% perturbation scenarios.

The equal-weight scenario shows a slightly lower top 10 overlap ratio because it substantially increases the relative importance of aviation transport compared with the baseline scenario. Nevertheless, its Spearman coefficient remains high at 0.9556, suggesting that the overall ranking pattern remains stable. Under the ±20% perturbation scenarios, the Spearman coefficients are all above 0.99 and the top 10 overlap ratios remain 9/10, indicating that moderate increases or decreases in the importance of railway, highway, or aviation transport do not substantially alter the main accessibility pattern. These results suggest that the integrated accessibility conclusions are robust to reasonable changes in modal weights and are not an artifact of the baseline weighting scheme. Detailed top 10 rankings and the ranking changes in representative high-accessibility nodes are provided in Table A2 and Table A3.

## 4. Discussion

The Western China–Central Asia cross-border transportation network is a complex system shaped by the joint effects of node connectivity, community organization, and accessibility performance. Traditional centrality indicators can identify key hubs and their network control capacity, while structural entropy and modal diversity entropy further reveal whether network connections are evenly distributed and whether node-level transportation modes are diversified. Combining entropy indicators with community structure and accessibility results helps provide a more comprehensive explanation of the operating mechanism and development bottlenecks of the cross-border transportation network.

The theoretical contribution of this study should therefore be understood as framework-level integration rather than formula-level innovation. Structural entropy and modal diversity entropy are established entropy-based measures, but their combined use in a cross-border multimodal transportation network remains limited. By linking entropy-based structural diagnosis with community organization and accessibility performance, this study provides an integrated structure–organization–function explanation of how hub concentration, modal imbalance, and accessibility differentiation jointly shape cross-border transportation performance.

### 4.1. Structure–Organization–Function Coupling Relationship in the Cross-Border Transportation Network

Node centrality and the balance of connection distribution jointly determine the agglomeration and segmentation characteristics of the cross-border transportation network. High-centrality nodes such as Xi’an, Urumqi, Almaty, and Tashkent form strong connectivity advantages and network control capacity through multimodal superposition.

The structural entropy results further show that different transportation modes contribute differently to this pattern. Under the original network-specific setting, the integrated transportation network shows relatively low structural entropy, indicating that core hubs still maintain strong comprehensive connection advantages after multimodal superposition. However, the common-node sensitivity analysis shows that the integrated network has higher structural entropy than the railway and aviation networks but lower structural entropy than the highway network. This suggests that multimodal integration does not simply intensify hub concentration. Rather, it simultaneously reinforces the role of core hubs and expands multimodal coverage across a broader set of nodes. Therefore, the integrated network should be understood as reflecting the coexistence of core-hub agglomeration and multimodal coverage expansion. These entropy-based findings provide topological diagnostics of connection distribution, but they should not be interpreted as direct evidence of actual transport capacity, service frequency, or passenger and freight flows.

Community structure extends these topological differences to the organizational level. The railway and highway networks are more strongly segmented by terrain, national borders, and corridor layout, whereas the aviation network shows stronger cross-regional connectivity. This produces an overall pattern of local cohesion and global segmentation, which further shapes the accessibility pattern of Western China core and Central Asian periphery. The Louvain resolution sensitivity analysis and modal-weight sensitivity analysis both confirm that these organizational and accessibility patterns are robust rather than artifacts of specific parameter settings.

A further limitation concerns the degree-based nature of the entropy indicators. Both structural entropy and modal diversity entropy in the main analysis are calculated using node degrees, namely the number of connections. This is useful for measuring topological balance, but it cannot fully reflect actual transport capacity, service frequency, passenger and freight flows, or operational efficiency. For example, a node with many low-frequency highway links may appear structurally balanced in a degree-based network, whereas a high-capacity railway hub with fewer but more efficient connections may be underestimated.

To partially address this limitation, this study conducted a travel-time-weighted entropy sensitivity analysis using inverse travel time as the connection strength. The weighted results show that the main conclusions remain generally stable: the highway network still has the most balanced connection distribution, the aviation network remains relatively concentrated around core airport cities, and the integrated transportation network continues to reflect the coexistence of core-hub agglomeration and multimodal coverage expansion. However, travel-time weighting still cannot fully replace operational data such as passenger volume, freight volume, train frequency, flight frequency, route capacity, or border-crossing efficiency. Therefore, the entropy results should be interpreted as a combination of topological diagnosis and travel-time-based sensitivity analysis, rather than as direct measurements of actual transport demand or service supply.

### 4.2. Differences in Node Modal Diversity and Multimodal Coordination

Table 7 shows that functional differences among cross-border transportation nodes depend not only on degree and centrality but are also closely related to the balance of modal configuration. Integrated core nodes have strong network control capacity, but some still show a bias toward aviation or railway transportation. Although multimodally balanced nodes may not have the highest connectivity, they have the potential to undertake regional transfer and modal conversion functions. Cross-border gateway nodes occupy prominent structural positions, but their insufficient modal redundancy makes it difficult to fully transform their corridor advantages into integrated accessibility advantages. Single-mode dependence nodes exhibit weaker transport substitutability and potential resilience. Therefore, the evaluation of cross-border transportation nodes should comprehensively consider multiple dimensions, including centrality, modal balance, and functional performance.

### 4.3. Development Bottlenecks and Optimization Strategies of the Cross-Border Transportation Network

The combined results of structural entropy, modal diversity entropy, community structure, and accessibility show that the main bottlenecks are not limited to infrastructure insufficiency. They also arise from core-hub overconcentration, imbalanced multimodal configuration, and insufficient cross-border organizational efficiency. Accordingly, three optimization directions are proposed.

First, the comprehensive service capacity of core hubs such as Xi’an, Urumqi, Almaty, and Tashkent should be strengthened. At the same time, secondary nodes and regional transfer nodes with relatively high modal diversity entropy should be cultivated to form a multilevel network jointly supported by core hubs, secondary hubs, and peripheral nodes, thereby reducing excessive dependence on a few core nodes.

Second, for nodes with low modal entropy and single-mode dependence, shortcomings in railway, highway, or aviation connections should be addressed, and transfer, transshipment, and collection-distribution capacities among different transportation modes should be improved. Cross-border gateway nodes such as Alashankou and Khorgos should be strengthened in terms of customs clearance organization and multimodal transport connections. The construction of digital ports, single-window systems, and transport information-sharing platforms should also be promoted to reduce the boundary friction caused by national borders and institutional differences.

Third, investment in transportation infrastructure should be increased in inland Central Asian areas, mountainous cities, and peripheral nodes to enhance the coverage, transport redundancy, and regional coordination capacity of the cross-border network.

## 5. Conclusions and Prospects

### 5.1. Conclusions

By constructing a model of the Western China–Central Asia cross-border integrated transportation network, this study integrates complex network analysis, established entropy-based indicators, community detection, and accessibility assessment to examine the network from three dimensions: structure, organization, and function. The main conclusions are as follows:

First, the cross-border transportation network exhibits significant structural heterogeneity and core–periphery differentiation. Nodes such as Xi’an, Urumqi, Almaty, and Tashkent have advantages in degree, betweenness centrality, and accessibility, making them core hubs for cross-regional factor flows and transportation organization. Although cross-border gateway nodes such as Alashankou and Khorgos have limited connectivity, they play a strong intermediary role in cross-border corridors.

Second, the structural entropy results show that the highway network has the most balanced connection distribution, the railway network exhibits corridor-based agglomeration, and the aviation network shows a strong hub-and-spoke structure. The common-node and travel-time-weighted sensitivity analyses further indicate that the integrated transportation network reflects the coexistence of core-hub agglomeration and multimodal coverage expansion, rather than a simple strengthening of connection concentration.

Third, modal diversity entropy reveals an inconsistency between node connectivity and the balance of transportation modes. Some medium-sized nodes have strong multimodal coordination potential because their railway, highway, and aviation configurations are relatively balanced. However, some core hubs and cross-border gateway nodes still show modal bias or missing transportation modes. Single-mode dependence nodes account for 41.8% of all 376 city-level nodes, indicating that modal redundancy and transport substitutability remain insufficient. This modal imbalance remains evident after travel-time-weighted modal diversity entropy is considered.

Fourth, the community detection results show that the cross-border transportation network has clear modular segmentation characteristics. The railway and highway networks are strongly affected by national borders, terrain, and corridor layout, with dense intra-community connections but weak links between cross-border communities. The aviation network has relatively stronger cross-regional integration capacity. Although the integrated transportation network strengthens connections within core areas, it still presents an organizational pattern of local cohesion and global segmentation. The resolution sensitivity analysis further indicates that this community organization pattern remains robust under different resolution settings.

Fifth, the accessibility analysis confirms the spatial differentiation pattern of Western China core and Central Asian periphery. Nodes such as Xi’an, Lanzhou, Urumqi, Almaty, and Tashkent form strong accessibility advantages through multimodal superposition, whereas peripheral areas in Central Asia, mountainous cities, and some border ports still face accessibility shortcomings. Overall, improving the performance of the cross-border transportation network cannot rely solely on increasing the number of corridors. It also requires enhancing structural balance, multimodal coordination, and cross-border organizational efficiency.

These findings suggest that future optimization should not focus solely on adding new corridors. Greater attention should be paid to strengthening the coordinated division of labor between core hubs and secondary nodes, improving modal redundancy at single-mode dependence nodes, and enhancing customs clearance, information sharing, and multimodal transport services at border ports and cross-border corridors.

### 5.2. Prospects

Based on the research limitations of this study and the practical needs of cross-border transport development between Western China and Central Asia, future research can be further advanced in the following three aspects.

First, research dimensions and data precision should be expanded. This study mainly conducts a static analysis based on data on physical transport connectivity and travel time in 2024. Future research could incorporate traffic flow data and time-series data to develop flow-weighted structural entropy and dynamic modal diversity entropy indicators, thereby revealing the evolutionary characteristics of cross-border transport networks.

Second, cross-border coordination mechanisms should be further examined. Although this study includes a scenario-based modal-weight sensitivity analysis, the weighting scheme cannot fully reflect actual passenger and freight flows, transport capacity, service frequency, or border-crossing efficiency. Future research could incorporate these operational data to construct flow-weighted or capacity-weighted accessibility models.

Third, this study has not yet conducted dynamic simulations under scenarios such as node failure, corridor disruption, and geopolitical risk shocks. Future research could set up scenarios such as random failures, targeted attacks, and disruptions of key border ports, and compare changes in network efficiency, the largest connected subgraph, and accessibility, thereby providing a more in-depth assessment of the vulnerability and resilience of the cross-border transport network between Western China and Central Asia.

## Figures and Tables

**Table 1:** Comparison of normalized structural entropy under original and common-node setting.

Network	Number of Nodes	Degree Sum	Original Normalized Structural Entropy	Common Node	Common-Node Normalized Structural Entropy
Railway Network	247	1042	0.9216	376	0.8563
Aviation Network	119	2028	0.8777	376	0.7074
Highway Network	320	981	0.9795	376	0.9529
Integrated Transportation Network	376	4051	0.8718	376	0.8718

**Table 2:** Modal diversity entropy and transportation mode composition of representative nodes.

Type	Representative Node	Highway Degree	Railway Degree	Aviation Degree	Total Degree	Modal Entropy	Normalized Modal Entropy
High Modal Balance Type	Wuhai	5	4	5	14	1.0934	0.9952
	Wuzhou	4	4	5	13	1.0928	0.9947
	Samarkand	4	3	4	11	1.0901	0.9922
Medium–High Modal Coordination Type	Xining	5	17	42	64	0.8277	0.7534
	Nanning	7	21	35	63	0.9369	0.8528
	Yinchuan	4	13	37	54	0.7947	0.7233
Medium–Low Modal Coordination Type	Garzê Tibetan Autonomous Prefecture	6	0	3	9	0.6365	0.5794
	Wenshan	3	0	6	9	0.6365	0.5794
	Baoji	4	8	0	12	0.6365	0.5794
Low Modal Diversity Type	Chengdu	6	18	167	191	0.4486	0.4084
	Dunhuang	0	1	19	20	0.1985	0.1807
	Xishuangbanna	0	1	41	42	0.1125	0.1024
Single-Mode Dependence Type	Qal’ai Khumb	2	0	0	2	0	0
	Khorog	3	0	0	3	0	0
	Murghab	3	0	0	3	0	0

**Table 3:** Sensitivity comparison between degree-based and travel-time-weighted entropy indicators.

Indicator	Degree-Based Result	Travel-Time-Weighted Result
Railway structural entropy	0.8563	0.8444
Highway structural entropy	0.9529	0.9274
Aviation structural entropy	0.7074	0.7245
Integrated structural entropy	0.8718	0.9003
Mean normalized modal diversity entropy	0.3849	0.3812
Share of single-mode dependence nodes	41.8%	41.8%
Share of strongly imbalanced nodes	45.5%	47.9%

Note: Structural entropy values are common-node normalized values based on 376 city-level nodes. Strongly imbalanced nodes refer to nodes with normalized modal diversity entropy lower than 0.45.

**Table 4:** Resolution sensitivity analysis of Louvain community detection.

Network	γ = 0.5	γ = 0.8	γ = 1.0	γ = 1.2	γ = 1.5	γ = 2.0
Railway	23/0.670	14/0.693	12/0.673	11/0.693	10/0.685	9/0.668
Highway	20/0.811	16/0.915	13/0.823	12/0.822	10/0.807	9/0.797
Aviation	12/0.313	7/0.350	4/0.364	3/0.349	3/0.352	2/0.188
Integrated	21/0.641	14/0.679	12/0.668	10/0.678	9/0.632	7/0.628

Note: Each cell reports the number of communities and the modularity value.

**Table 5:** Weighting scenarios for integrated accessibility sensitivity analysis.

Scenario	Railway	Highway	Aviation	Purpose
S0 Baseline	0.450	0.400	0.150	Original setting
S1 Equal Weight	0.333	0.333	0.333	Neutral benchmark
S2 Railway +20%	0.540	0.335	0.125	Railway weight increased by 20%
S3 Railway −20%	0.360	0.465	0.175	Railway weight decreased by 20%
S4 Highway +20%	0.390	0.480	0.130	Highway weight increased by 20%
S5 Highway −20%	0.510	0.320	0.170	Highway weight decreased by 20%
S6 Aviation +20%	0.434	0.386	0.180	Aviation weight increased by 20%
S7 Aviation −20%	0.466	0.414	0.120	Aviation weight decreased by 20%

**Table 6:** Robustness of integrated accessibility rankings under alternative weighting scenarios.

Scenario	Spearman Correlation with S0	Top 10 Overlap with S0	Top 10 Overlap Ratio
S1 Equal Weight	0.9556	8/10	0.80
S2 Railway +20%	0.9930	9/10	0.90
S3 Railway −20%	0.9919	9/10	0.90
S4 Highway +20%	0.9916	9/10	0.90
S5 Highway −20%	0.9924	9/10	0.90
S6 Aviation +20%	0.9977	9/10	0.90
S7 Aviation −20%	0.9964	9/10	0.90

**Table 7:** Cross-border transportation node types and structure–modal–function characteristics.

Node Type	Typical Nodes	Structural Characteristics	Modal Diversity Entropy Characteristics	Organizational Function Performance
Integrated Core Nodes	Xi’an Urumqi Almaty Tashkent	High connectivity and centrality; strong network control	Diverse transport modes; aviation or railway transport dominates at some nodes	High accessibility and strong regional influence, but with notable hub-dependence risk
Multimodal Balanced Nodes	Wuhai Wuzhou Samarkand	Moderate connectivity; balanced configuration	Normalized modal diversity entropy close to 1; strong multimodal coordination potential	Strong potential for regional transfer and modal interchange
Cross-Border Gateway Nodes	Alashankou Khorgos	Limited connectivity; strong intermediary role	Imbalanced modal structure; insufficient modal redundancy	Prominent structural role but limited accessibility advantage
Single-Mode Peripheral Nodes	Qal’ai Khumb Khorog Murghab	Peripheral position; few connecting routes	Modal diversity entropy of 0; single-mode dependence	Weak external links, low accessibility, limited transport substitutability and resilience

## Data Availability

The raw data used in this study were obtained from publicly available and platform-based sources, including OpenStreetMap, the National Geomatics Center of China, Tianditu, Baidu Maps, Amap, Google Maps, China Railway 12306, and Amap travel-time services. The processed datasets generated during the current study are available from the corresponding author upon reasonable request.

## Appendix A

**Table A1:** Community membership of representative hub and corridor nodes under different resolution values.

Type	Hub City	Layer	Label	γ = 0.5	γ = 0.8	γ = 1.0	γ = 1.2	γ = 1.5	γ = 2.0
Railway	Xi’an	City node	Xi’an	C7 (n = 10)	C4 (n = 38)	C4 (n = 55)	C2 (n = 56)	C0 (n = 56)	C2 (n = 68)
	Urumqi	City node	Urumqi	C4 (n = 23)	C4 (n = 38)	C2 (n = 35)	C2 (n = 56)	C0 (n = 56)	C2 (n = 68)
	Lanzhou	City node	Lanzhou	C4 (n = 23)	C4 (n = 38)	C2 (n = 35)	C2 (n = 56)	C0 (n = 56)	C2 (n = 68)
	Tashkent	City node	Tashkent	C12 (n = 15)	C12 (n = 15)	C10 (n = 45)	C5 (n = 45)	C6 (n = 15)	C5 (n = 15)
	Almaty	City node	Almaty	C17 (n = 21)	C9 (n = 30)	C6 (n = 30)	C7 (n = 32)	C8 (n = 50)	C7 (n = 50)
	Shymkent	City node	Shymkent	C17 (n = 21)	C9 (n = 30)	C6 (n = 30)	C7 (n = 32)	C8 (n = 50)	C7 (n = 50)
Highway	Xi’an	City node	Xi’an	C13 (n = 18)	C7 (n = 18)	C9 (n = 35)	C7 (n = 26)	C6 (n = 32)	C6 (n = 52)
	Urumqi	City node	Urumqi	C10 (n = 19)	C15 (n = 36)	C6 (n = 34)	C11 (n = 33)	C1 (n = 35)	C7 (n = 23)
	Lanzhou	City node	Lanzhou	C12 (n = 14)	C12 (n = 27)	C9 (n = 35)	C7 (n = 26)	C6 (n = 32)	C6 (n = 52)
	Tashkent	City node	Toshkent	C6 (n = 22)	C5 (n = 18)	C5 (n = 20)	C0 (n = 20)	C3 (n = 23)	C3 (n = 42)
	Almaty	City node	Almaty	C5 (n = 18)	C0 (n = 17)	C6 (n = 34)	C2 (n = 19)	C3 (n = 23)	C3 (n = 42)
	Shymkent	City node	Shymkent	C6 (n = 22)	C5 (n = 18)	C3 (n = 22)	C0 (n = 20)	C4 (n = 46)	C3 (n = 42)
Aviation	Xi’an	Airport node	Xi’an	C0 (n = 9)	C0 (n = 17)	C0 (n = 36)	C0 (n = 75)	C1 (n = 61)	C0 (n = 98)
	Urumqi	Airport node	Urumqit	C6 (n = 4)	C5 (n = 17)	C2 (n = 21)	C1 (n = 20)	C0 (n = 34)	C0 (n = 98)
	Lanzhou	Airport node	Lanzhou	C1 (n = 7)	C3 (n = 28)	C2 (n = 21)	C1 (n = 20)	C0 (n = 34)	C0 (n = 98)
	Tashkent	Airport node	Tashkent	C10 (n = 12)	C6 (n = 32)	C3 (n = 32)	C2 (n = 32)	C2 (n = 32)	C1 (n = 29)
	Almaty	Airport node	Almaty	C11 (n = 20)	C6 (n = 32)	C3 (n = 32)	C2 (n = 32)	C2 (n = 32)	C1 (n = 29)
	Shymkent	Airport node	Shymkent	C11 (n = 20)	C6 (n = 32)	C3 (n = 32)	C2 (n = 32)	C2 (n = 32)	C1 (n = 29)
Integrated	Xi’an	Highway layer	H-Xi’an	C9 (n = 30)	C6 (n = 95)	C7 (n = 102)	C5 (n = 103)	C2 (n = 131)	C3 (n = 144)
	Xi’an	Railway layer	T-Xi’an	C17 (n = 48)	C6 (n = 95)	C7 (n = 102)	C5 (n = 103)	C2 (n = 131)	C3 (n = 144)
	Urumqi	Highway layer	H-Urumqi	C6 (n = 48)	C2 (n = 76)	C4 (n = 71)	C4 (n = 67)	C5 (n = 64)	C6 (n = 154)
	Urumqi	Railway layer	T-Urumqi	C17 (n = 48)	C6 (n = 95)	C7 (n = 102)	C5 (n = 103)	C2 (n = 131)	C3 (n = 144)
	Lanzhou	Highway layer	H-Lanzhou	C9 (n = 30)	C6 (n = 95)	C7 (n = 102)	C5 (n = 103)	C2 (n = 131)	C3 (n = 144)
	Lanzhou	Railway layer	T-Lanzhou	C17 (n = 48)	C6 (n = 95)	C7 (n = 102)	C5 (n = 103)	C2 (n = 131)	C3 (n = 144)
	Tashkent	Highway layer	H-Toshkent	C0 (n = 34)	C11 (n = 48)	C2 (n = 143)	C3 (n = 126)	C8 (n = 119)	C4 (n = 218)
	Tashkent	Railway layer	T-Tashkent	C0 (n = 34)	C11 (n = 48)	C2 (n = 143)	C3 (n = 126)	C8 (n = 119)	C4 (n = 218)
	Almaty	Highway layer	H-Almaty	C5 (n = 38)	C2 (n = 76)	C4 (n = 71)	C4 (n = 67)	C5 (n = 64)	C4 (n = 218)
	Almaty	Railway layer	T-Almaty	C3 (n = 26)	C1 (n = 74)	C2 (n = 143)	C3 (n = 126)	C0 (n = 113)	C4 (n = 218)
	Shymkent	Highway layer	H-Shymkent	C0 (n = 34)	C11 (n = 48)	C2 (n = 143)	C3 (n = 126)	C8 (n = 119)	C4 (n = 218)
	Shymkent	Railway layer	T-Shymkent	C3 (n = 26)	C1 (n = 74)	C2 (n = 143)	C3 (n = 126)	C0 (n = 113)	C4 (n = 218)
	Xi’an	Airport layer	A-Xi’an	C20 (n = 27)	C13 (n = 82)	C10 (n = 81)	C9 (n = 102)	C8 (n = 119)	C6 (n = 154)
	Urumqi	Airport layer	A-Urumqi	C19 (n = 33)	C13 (n = 82)	C10 (n = 81)	C9 (n = 102)	C8 (n = 119)	C6 (n = 154)
	Lanzhou	Airport layer	A-Lanzhou	C20 (n = 27)	C13 (n = 82)	C10 (n = 81)	C9 (n = 102)	C8 (n = 119)	C6 (n = 154)
	Tashkent	Airport layer	A-Tashkent	C0 (n = 34)	C11 (n = 48)	C2 (n = 143)	C3 (n = 126)	C8 (n = 119)	C4 (n = 218)
	Almaty	Airport layer	A-Almaty	C0 (n = 34)	C11 (n = 48)	C2 (n = 143)	C3 (n = 126)	C8 (n = 119)	C4 (n = 218)
	Shymkent	Airport layer	A-Shymkent	C0 (n = 34)	C11 (n = 48)	C2 (n = 143)	C3 (n = 126)	C8 (n = 119)	C4 (n = 218)

Note: Community class IDs are generated separately for each resolution value and are not directly comparable across different resolutions. The table is used to indicate the community membership of representative hub and corridor nodes under alternative resolution settings. In the integrated multilayer network, H-, T-, and A- represent highway, railway, and aviation layers, respectively.

**Table A2:** Top 10 nodes under different weighting scenarios.

RANK	S0 Baseline	S1 Equal Weight	S2 Railway +20%	S3 Railway −20%	S4 Highway +20%	S5 Highway −20%	S6 Aviation +20%	S7 Aviation −20%
1	Xi’an	Xi’an	Xi’an	Xi’an	Xi’an	Xi’an	Xi’an	Xi’an
2	Chengdu	Chengdu	Chengdu	Chengdu	Chengdu	Chengdu	Chengdu	Chengdu
3	Nanning	Guiyang	Nanning	Nanning	Nanning	Nanning	Nanning	Nanning
4	Yinchuan	Chongqing	Yinchuan	Yinchuan	Yinchuan	Kunming	Kunming	Yinchuan
5	Kunming	Kunming	Kunming	Guiyang	Kunming	Yinchuan	Guiyang	Kunming
6	Guiyang	Urumqi	Guiyang	Kunming	Guiyang	Guiyang	Yinchuan	Guiyang
7	Urumqi	Nanning	Xining	Urumqi	Urumqi	Urumqi	Urumqi	Xining
8	Xining	Yinchuan	Urumqi	Xining	Xining	Xining	Xining	Urumqi
9	Tashkent	Zunyi	Tashkent	Chongqing	Tashkent	Tashkent	Chongqing	Tashkent
10	Lanzhou	Xining	Banan District	Tashkent	Banan District	Chongqing	Tashkent	Banan District

**Table A3:** Ranking changes in representative high-accessibility nodes under alternative weighting scenarios.

City	S0 Rank	S1 Rank	S2 Rank	S3 Rank	S4 Rank	S5 Rank	S6 Rank	S7 Rank
Xi’an	1	1	1	1	1	1	1	1
Chengdu	2	2	2	2	2	2	2	2
Nanning	3	7	3	3	3	3	3	3
Yinchuan	4	8	4	4	4	5	6	4
Urumqi	7	6	8	7	7	7	7	8
Tashkent	9	12	9	10	9	9	10	9
Almaty	23	19	26	22	23	23	23	23
Shymkent	30	24	34	30	35	29	30	34
Fergana	46	57	57	37	33	58	50	42
Andijan	47	74	51	41	36	56	54	41

Note: S0 = baseline; S1 = equal − weight; S2 = railway + 20%; S3 = railway − 20%; S4 = highway + 20%; S5 = highway − 20%; S6 = aviation + 20%; S7 = aviation − 20%.
